# Characteristics of 419 patients with acquired middle ear cholesteatoma^☆^

**DOI:** 10.1016/j.bjorl.2016.02.013

**Published:** 2016-05-03

**Authors:** Letícia Petersen Schmidt Rosito, Maurício Noschang Lopes da Silva, Fábio André Selaimen, Yuri Petermann Jung, Marcos Guilherme Tibes Pauletti, Larissa Petermann Jung, Luiza Alexi Freitas, Sady Selaimen da Costa

**Affiliations:** a Hospital de Clínicas de Porto Alegre, Porto Alegre, RS, Brazil; b Universidade Federal do Rio Grande do Sul (UFRGS), Porto Alegre, RS, Brazil; c Universidade Federal do Rio Grande do Sul (UFRGS), Faculdade de Medicina, Porto Alegre, RS, Brazil; d Universidade Federal do Rio Grande do Sul (UFRGS), Faculdade de Medicina, Departamento de Oftalmologia e Otorrinolaringologia, Porto Alegre, RS, Brazil

**Keywords:** Cholesteatoma, Middle ear, Bilateral, Classification, Surgery, Colesteatoma, Orelha média, Bilateral, Classificação, Cirurgia

## Abstract

**Introduction:**

Cholesteatoma is a destructive lesion that can result in life-threatening complications. Typically, it presents with hypoacusis and continuous otorrhea as symptoms. Because it is a rare disease, there are few studies in Brazil describing the characteristics of patients with the disease.

**Objective:**

This study aimed to determine the prevalence of cholesteatoma in patients with chronic otitis media and describe clinical, audiological and surgical characteristics of patients with acquired middle ear cholesteatoma treated at a referral hospital in the public health system.

**Methods:**

Cross-sectional and prospective cohort study, including 1710 patients with chronic otitis media, treated between August 2000 and June 2015, without prior surgery. Detailed clinical history, videotoscopy, and audiometry were performed, in addition to review of medical records to search for surgical data. Cholesteatomas were classified according to their route of formation.

**Results:**

Of the patients with chronic otitis media, 419 (24.5%) had cholesteatoma; mean age of 34.49 years; 53.5% female and 63.8% adults. Bilateral cholesteatoma was observed in 17.1%. Anterior epitympanic cholesteatoma corresponded to 1.9%; posterior epitympanic, 32.9%; posterior mesotympanic, 33.7%; two routes, 14.8%; and indeterminate, 16.7%. The mean air-bone gap was 29.84 dB and did not differ between routes of formation. There were no correlations between gap size and patient age or duration of symptoms. Of the surgical cases, 16.8% underwent closed tympanomastoidectomy and 75.2% open tympanomastoidectomy.

**Conclusion:**

The prevalence of cholesteatoma in patients with chronic otitis media was 24.5% and it was more common in adults than in children. Posterior mesotympanic cholesteatoma was more frequent, with no difference in mean air-bone gap between the different routes of formation. In patients undergoing surgery, open tympanomastoidectomy was the procedure most frequently chosen.

## Resumo

**Introdução:**

Colesteatoma é uma lesão destrutiva que pode determinar complicações potencialmente letais. Tipicamente, apresenta hipoacusia e otorreia contínua como sintomas. Por ser uma doença rara, existem poucos estudos no Brasil que descrevam as características dos pacientes portadores.

**Objetivo:**

O presente estudo teve como objetivos determinar a prevalência de colesteatoma entre os pacientes com otite média crônica (OMC) e descrever características clínicas, audiológicas e cirúrgicas dos pacientes com colesteatoma adquirido de orelha média atendidos em um hospital de referência do sistema público de saúde.

**Método:**

Estudo transversal e de coorte prospectivo, incluindo 1.710 pacientes com OMC, atendidos entre agosto de 2000 e junho de 2015, sem tratamento cirúrgico prévio. Foi realizada anamnese detalhada, videotoscopia e audiometria, além de revisão de prontuários para busca de dados cirúrgicos. Os colesteatomas foram classificados conforme sua via de formação.

**Resultados:**

Dos pacientes com otite média crônica, 419 (24,5%) apresentaram colesteatoma. Média de idade de 34,49 anos; 53,5% do sexo feminino e 63,8% adultos. Colesteatoma foi observado bilateralmente em 17,1%. Os epitimpânicos anteriores corresponderam a 1,9%; os epitimpânicos posteriores a 32,9%; os mesotimpânicos posteriores a 33,7%; duas vias a 14,8% e indeterminados a 16,7%. A média tritonal dos *gaps* aero-ósseos foi de 29,84 dB e não diferiu entre as vias de formação. Não foram observadas correlações entre tamanho do *gap* e idade do paciente ou duração dos sintomas. Dos pacientes operados, 16,8% foram submetidos a timpanomastoidectomia fechada e 75,2% a timpanomastoidectomia aberta.

**Conclusão:**

A prevalência de colesteatoma em pacientes com otite média crônica foi de 24,5%, e foi mais frequente em adultos do que em crianças. Os mesotimpânicos posteriores foram mais frequentes, não sendo observada diferença na média dos *gaps* aero-ósseos entre diferentes vias de formação. Nos pacientes submetidos a cirurgia, a timpanomastoidectomia aberta foi o procedimento escolhido.

## Introduction

Cholesteatoma is considered a benign epithelial lesion with a gradual and destructive expansion, which affects the ear canal and adjacent structures.^1^ Epithelial accumulation and bone erosion, characteristic of the disease, typically result in continuous otorrhea and hypoacusis. With the progression of cholesteatoma, there may also be involvement of the inner ear^2^, ^3^ and facial nerve, in addition to serious complications such as meningitis and brain abscess.^4^

The estimated incidence in the general population is 3.7–13.9/100,000.^5^, ^6^ This incidence is lower in children (3/100,000) than in adults (9/100,000).^7^ Because it is a rare disease, epidemiological studies, especially in Brazil, are scarce. This lack of data makes it impossible to compare studies performed in Brazil with others, as it is not known whether the populations are really similar.

The objectives of this study were to determine the prevalence of cholesteatoma in patients with chronic otitis media (COM); describe the clinical and audiological characteristics of patients with cholesteatoma; and verify the surgical techniques most used in a referral hospital in the public health system.

## Methods

This was a cross-sectional and prospective cohort study, including 1710 consecutive patients with COM attended to during the period from August 2000 to June 2015 at the Clinic of Chronic Otitis Media of the Hospital de Clínicas de Porto Alegre.

The inclusion criterion for follow-up was patients with COM with cholesteatoma diagnosed after videotoscopy. Exclusion criteria were history of any previous ear surgery, except tympanostomy for placement of tympanostomy tubes, and inability to properly clean the ears and/or to perform videotoscopy for proper documentation.

At the first assessment, a detailed medical history was taken, as well as a clinical exam. After careful cleaning of the ears, videotoscopy was performed with a fiber optic zero degree and 4 mm telescope (Karl Stortz). Both ears were recorded sequentially using the Cyberlink Power Director software (version 7, 2008). After the medical history and physical examination, the patients underwent pure tone audiometry, using the audiometer Interacoustics AD 27 with supra-aural phones TDH-39, for determining the air conduction and bone conduction thresholds, and air-bone gap. The gap was calculated from the difference between the air and bone conduction thresholds. Broadband masking noise was used when necessary. In small children, playful conditioned audiometry was used with supra-aural phones. When necessary, audiometry was completed after two sessions for confirmation of the obtained thresholds. Airway, bone conduction, and gap were described by tritonal mean, calculated by the average of thresholds at 500, 1000, and 2000 Hz, representing the speech recognition area.

The videotoscopies were independently reviewed during a clinical meeting. For the evaluation of the images, specific protocols were used in a systematic manner, in accordance with the definitions described below by the senior investigator.

Cholesteatomas were then classified according to the modified Jackler classification,^8^ according to their route of formation:

1. Anterior epitympanic: when originated anteriorly to the head of the malleus.
2. Posterior epitympanic: when originated exclusively in the pars flaccida.
3. Posterior mesotympanic: when originated exclusively in the posterior-superior quadrant of the pars tensa.
4. Two routes: when involved both the pars flaccida and pars tensa.
5. Undetermined: when the cholesteatoma route of formation could not be determined by videotoscopy image.

Patients aged under 18 were considered as children, according to the criteria of the World Health Organization.

Medical records and surgical descriptions of patients who had undergone surgery were reviewed.

The Research and Graduate Group of our institution approved the project in its ethical and scientific aspects (Protocol No. 14918). All patients included in the study had previously signed an informed consent authorizing the use of their data anonymously in this scientific study.

Data were tabulated and analyzed using the SPSS software program (version 20). Statistical analysis was performed using the Mann–Whitney test to evaluate the difference between the duration of the disease, and Fisher's exact test for the difference in prevalence of palatal defects among children and adults. ANOVA test was used to compare the mean tritonal gaps between the different routes of cholesteatoma formation. Correlations were performed using the Spearman test. *p*-values < 0.05 were considered to be statistically significant.

## Results

Of the 1710 patients with COM evaluated in the study, cholesteatoma was present in 419 (24.5%). The mean age of patients was 34.49; standard deviation (SD) 19.8, and 53.5% were female. White patients accounted for 86.5% of the study population and 5.9% were black. Adults corresponded to 63.8%. Cholesteatoma was identified in the right ear was in 234 patients (55.8%).

The prevalence of palate defects in this population was 4.3%. There was no difference in prevalence of palatal defects between children and adults (3.4% *vs*. 4.9%, respectively; *p* = 0.59).

The average time from the onset of symptoms to the assessment was 13.73 years (SD = 14.43). In children, the average was 6.39 years and median of 6 years, in adults it was of 17.63 years and median of 12, respectively. There was a statistically significant difference between groups (*p* < 0.001).

**Figure 1:**
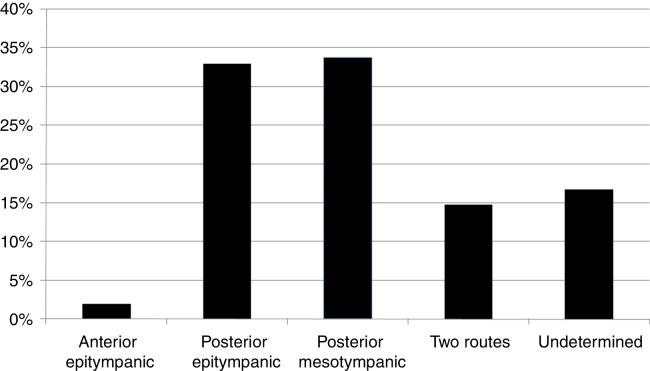
Prevalence of cholesteatomas according to their growth pattern.

The prevalence of the different routes of cholesteatoma formation in this population is shown in Fig. 1. There was no difference between the duration of symptoms and the route of formation (*p* = 0.28).

In the contralateral ear evaluation, only 150 of them (36.1%) were normal and cholesteatoma was identified in 71 (17.1%). The duration of symptoms was longer in patients with changes in the contralateral ear than in those with normal contralateral ear (mean of 14.99 and 11.69, respectively; *p* = 0.007). There was no difference in the prevalence of cholesteatoma in the contralateral ear between children and adults (*p* = 0.20) and between patients with and without palate malformation (*p* = 0.19).

**Figure 2:**
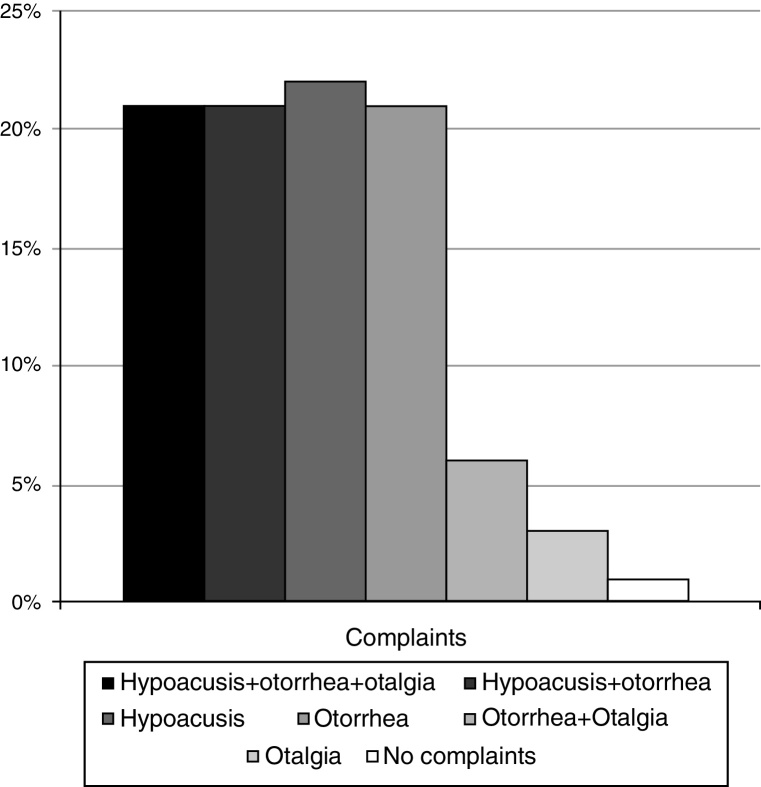
Frequency of the principle complaints of patients with cholesteatoma at the time of first evaluation.

The main complaints of patients at the time of the first evaluation in this service are shown in Fig. 2. Hypoacusis, with or without otorrhea, was the main complaint of 84.4% of the study population, and otorrhea was observed in 87%. There were no differences regarding the main complaint when we compared cholesteatomas classified by the route of formation (*p* = 0.27).

**Figure 3:**
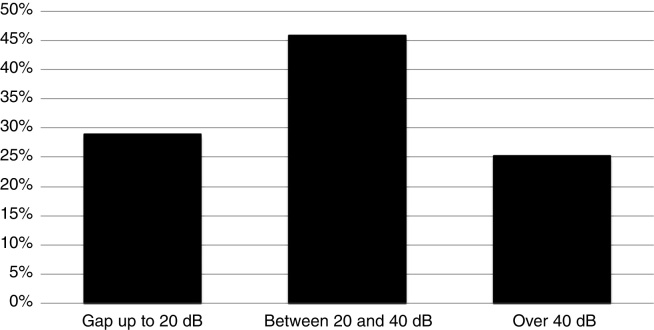
Prevalence of the air-bone difference sizes in tritonal means.

**Figure 4:**
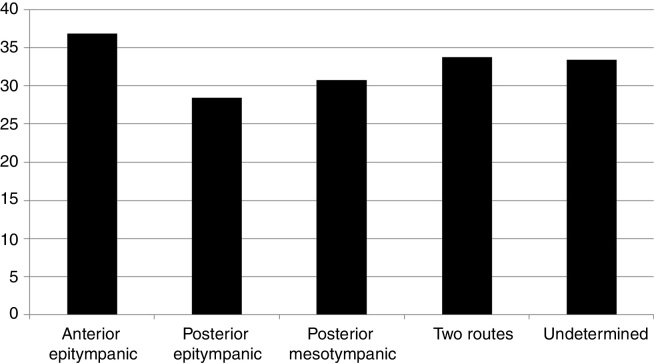
Comparison of tritonal means of air-bone gap (dB) between the different routes of cholesteatoma formation.

Among the evaluated patients, 92.41% underwent audiometry. Regarding bone conduction and airway thresholds, the tritonal mean was 17.08 (SD) 16.16 dB and 46.35 (SD) 22.34 dB, respectively. Regarding gap sizes, the tritonal mean was 29.84 (SD) 13.61. The prevalence of air-bone gap sizes in tritonal means is shown in Fig. 3. There was no correlation between gap size and age of the patient at the time of evaluation (*R* = 0.03; *p* = 0.55) or duration of symptoms (*R* = 0.08; *p* = 0.13). No differences were observed regarding gap tritonal mean between the different routes of cholesteatoma formation (*p* = 0.13), as shown in Fig. 4.

Among all the patients, 205 (48.9%) had not undergone surgery at the time of the study or had been lost at follow-up. Of the patients who underwent surgery, 17 (7.9%) underwent tympanoplasty; 36 (16.8%), closed tympanomastoidectomy; and 161 (75.2%), open tympanomastoidectomy. There was no difference in the surgical technique used in children and adults (*p* = 0.86).

## Discussion

Descriptive studies of the clinical characteristics of patients with cholesteatoma in Brazil are scarce. A previous study performed in the country, despite describing a large sample of patients, was based on data and medical records collected retrospectively, which can lead to several biases in the measurement.^6^ In the present study, all patients underwent the same medical history, and the ears were cleaned and prepared for proper videotoscopy. All images were taken with high quality to be subsequently evaluated by the same senior investigator. The use of videotoscopy therefore allows for better image definition, better documentation, and finally, a more detailed and accurate disease classification.

In the present study population, there was a higher prevalence of adults than children, which corroborates previous studies showing that the incidence of cholesteatomas is higher in adults.^7^ Hypoacusis and otorrhea were the main complaints of most patients, and 87% had otorrhea at the time of evaluation. Complaints of hearing loss were confirmed by audiometry, which showed that the vast majority of patients had conductive hearing loss with larger air-bone gap of 20 dB in speech recognition area.

The prevalence of bilateral cholesteatoma in this study was 17.1%; similar to previous studies.^9^ However, we found no differences in the prevalence of bilateral cholesteatoma between patients with and without palatal defects or between children and adults. These findings suggest that the mechanism involved in the pathogenesis of cholesteatoma, initiated by the Eustachian tube dysfunction, perpetuates with progressive retraction of the tympanic membrane and increase of gas exchange in the middle ear caused by the inflammatory process and, not necessarily, by the maintenance of the initial disorder.^10^ Thus, the high prevalence of bilaterality, even in patients without palate changes, can be understood since auditory tube disorders and secretory otitis media often affect both sides in most patients.^11^, ^12^

Posterior mesotympanic cholesteatomas were the most frequent in the present population, contrary to most studies reporting attic cholesteatoma as the most prevalent.^8^, ^13^ Furthermore, the documentation of anterior epitympanic cholesteatoma was very rare, and of cholesteatoma that developed concurrently in both the pars tensa and pars flaccida, which was called two routes, corresponded to 15% of cases. In about 15% of patients, it was not possible to determine accurately the route of cholesteatoma formation and therefore they were classified as undetermined.

The large average length of time between the onset of symptoms and the first evaluation demonstrates the difficulties patients with cholesteatoma have to access public service in Brazil. Although no correlation was observed between the duration of symptoms and the degree of conductive hearing loss, it was found that patients with changes in the contralateral ear had been symptomatic for a longer time. According to other authors data regarding the duration of disease, have poor reliability.^14^ They depend on patient's memory and often of previous symptoms of ear pain, otorrhea, or hypoacusis associated with a history of recurrent otitis media or chronic secretory otitis, which can be easily confused with the symptoms of cholesteatoma. A study by Aberg et al.^15^ compared the clinical symptoms of patients with cholesteatomatous and non-cholesteatomatous COM through a questionnaire between two groups and found no difference in symptoms. This may explain the large difference in the mean time from symptom onset between children and adults, as many adults indicate the beginning of their complaints in childhood.

Palate defects are a known risk factor for the development of cholesteatoma. Dominguez et al.^16^ estimated that the risk of cholesteatoma in a child with cleft palate is 2.6–9.2%. Spilsbrury et al.^17^ studied children who underwent placement of a ventilation tube at least once and found that 6.9% of the children with cleft palate developed cholesteatoma compared with 1.5% of those without cleft palate. Harris et al.^18^ found that 2.2% of patients undergoing palatoplasty between 5 and 18 years of age harbored cholesteatoma, an incidence 200 times greater than that observed in the general population. A previous study with this group showed a prevalence of 6.4% of cholesteatoma in patients with cleft lip and cleft palate or cleft palate alone.^19^ In the present study, a frequency of 3.8% of patients with palatal defects was observed, with no difference between children and adults, while Kemppainen et al.^5^ studied 500 patients with cholesteatoma and found a prevalence of 8%.

In the present series, 51.1% of patients underwent surgery in the study period. As this is a referral service for the treatment of COM, due to massive demand for surgery and low surgical availability, there is often a long delay from the first assessment to surgery. The long waiting time for the procedure and the major progression of the disease resulting from that delay, associated with the lack of adherence and follow-up difficulty of some patients, makes the open tympanomastoidectomy the surgical technique of choice. Currently in this service, open tympanomastoidectomy is indicated in the following situations: locally advanced disease, involvement of the posterolateral upper quadrants of the middle ear that prevents proper cleaning of the facial nerve recess and tympanic sinus; a sclerotic or eburnated mastoid; involvement of a single ear; and/or a contralateral ear with advanced cholesteatoma or previous cavity. The surgical technique indications were similar between children and adults. The present results are contrary to the international trend, currently more favorable to conservative surgeries, using closed tympanomastoidectomy or channel reconstruction technique.^20^, ^21^, ^22^

Epidemiological studies, such as the present, performed with a large number of patients, provide data on the Brazilian population, especially that population dependent on the public health system. Thus, knowing the clinical characteristics and peculiarities of the patients with cholesteatoma, it can be assessed whether the results of international studies can be reproduced and applied in the Brazilian population.

## Conclusion

The prevalence of cholesteatoma in patients with COM was 24.5%; it was more frequently found in adults than in children. Bilateral disease was found in 17.1% of patients with cholesteatoma. Hypoacusis, with or without otorrhea, was the main complaint, and the vast majority of patients had otorrhea at the time of the first evaluation. A higher prevalence of posterior mesotympanic cholesteatoma was observed in the present population. The frequency of palatal defects was 3.8%, similar in children and adults. Regarding air-bone audiometric gap sizes, the tritonal mean was 29.84 dB and there were no differences when comparing the different routes of cholesteatoma formation. In most patients undergoing surgery, open tympanomastoidectomy was the procedure of choice.

## Conflicts of interest

The authors declare no conflicts of interest.
